# Increasing the information provided by probabilistic sensitivity analysis: The relative density plot

**DOI:** 10.1186/s12962-020-00251-7

**Published:** 2020-11-30

**Authors:** Joost W. Geenen, Rick A. Vreman, Cornelis Boersma, Olaf H. Klungel, Anke M. Hövels, Renske M. T. Ten Ham

**Affiliations:** 1 Division of Pharmacoepidemiology and Clinical Pharmacology, Utrecht Institute for Pharmaceutical Sciences (UIPS), Utrecht University, Universiteitsweg 99, 3584CG Utrecht, The Netherlands; 2 The National Health Care Institute (ZIN), Diemen, The Netherlands; 3 Health-Ecore, 1e Hogeweg 196, Zeist, 3701 HL The Netherlands; 4 Division of Global Health, Department of Health Sciences, University of Groningen, University Medical Center Groningen, Antonius Deusinglaan 1, Groningen, 9713 AV The Netherlands

**Keywords:** Probabilistic sensitivity analysis, Relative density, Health economics, Modelling, Health technology assessment, Sensitivity analysis, Information, Knowledge, Uncertainty, Health

## Abstract

**Background:**

Results of probabilistic sensitivity analyses (PSA) are frequently visualized as a scatterplot, which is limited through overdrawing and a lack of insight in relative density. To overcome these limitations, we have developed the Relative Density plot (PSA-ReD).

**Methods:**

The PSA-ReD combines a density plot and a contour plot to visualize and quantify PSA results. Relative density, depicted using a color gradient, is transformed to a cumulative probability. Contours are then plotted over regions with a specific cumulative probability. We use two real-world case studies to demonstrate the value of the PSA-ReD plot.

**Results:**

The PSA-ReD method demonstrates proof-of-concept and feasibility. In the real-world case-studies, PSA-ReD provided additional visual information that could not be understood from the traditional scatterplot. High density areas were identified by color-coding and the contour plot allowed for quantification of PSA iterations within areas of the cost-effectiveness plane, diminishing overdrawing and putting infrequent iterations in perspective. Critically, the PSA-ReD plot informs modellers about non-linearities within their model.

**Conclusions:**

The PSA-ReD plot is easy to implement, presents more of the information enclosed in PSA data, and prevents inappropriate interpretation of PSA results. It gives modelers additional insight in model functioning and the distribution of uncertainty around the cost-effectiveness estimate.

## Background

Health economic models have become an integral part of healthcare decision making [1–4]. These models rely on input parameters associated with uncertainty which must be taken into account when calculating and presenting model results [5]. Deterministic and probabilistic sensitivity analyses (DSA and PSA) are systematic approaches that quantify the impact of uncertainties related to model inputs on the outcomes of the model [6]. In a PSA all input parameters are simultaneously varied along predefined ranges according to their specific distribution. The PSA has been the most prominent method to quantify the impact of combined uncertainty of all model input parameters [7]. Besides providing insight on uncertainty, a PSA also provides insights in the functionality of the model as it graphically conveys the relationship between the model structure, the input parameters and the outcomes. The output of a PSA is typically presented as a scatter plot in the cost-effectiveness plane (CE-plane) [8].

The traditional scatter plot is useful to quickly visualize the distribution of PSA results as well as the correlation between the cost and the effect measure of interest [9]. A critical aspect of the scatter plot is its ability to illustrate the distribution of PSA samples over the quadrants of the cost-effectiveness plane (i.e., increased Quality Adjusted Life Years [QALY] and increased costs or decreased QALYs and increased costs). The scatter plot itself is not the sole measure to quantify and interpret parameter uncertainty as, for example, the likelihood of cost-effectiveness is typically illustrated with a cost-effectiveness acceptability curve (CEAC). The PSA scatterplot is an intuitive, useful and usually mandatory figure in communication towards stakeholders, who might be less familiar with uncertainty analyses. To a modeler on the other hand, a scatterplot is a useful tool to quickly grasp the influence of changes to model structure and parameter distributions. Despite these advantages, the traditional scatter plot has two major limitations.

The first limitation is that in the traditional scatter plot, individual point estimates are overlapping in high density areas. This so-called overdrawing makes it hard to assess the relative density of point estimates in populous areas of the plot (Gleicher [10]). Second, due to difficulty in estimating this relative density, infrequent scenarios appear very prominent in the traditional figure. This may cause overestimation of the occurrence of these scenarios. These two limitations thus yield incorrect or incomplete insight in the relationship between the underlying model, its parameters and the outcomes.

To overcome these two limitations a novel presentation of the PSA scatter plot is desired. Increased computational power combined with increased popularity of open source software, such as R, provide the tools to improve the traditional PSA presentation (R Core team [11]). Two R-packages are frequently used to display PSA scatter plot results. The heemod package uses colored hexagons to display relative density which gives some information on overdrawing [12]. The Bayesian Cost-Effectiveness Analysis (BCEA) package by Baio et al. provides the tools to draw a contour plot using ellipses in discrete intervals. [13]. Both packages require the user to build a cost-effectiveness model using package specific syntax to be able to use and apply the package features. This requires extensive R-skills which can put-off users less familiar with the programming language. Additionally, the features available within these packages provide either a plot showing relative density (heemod) or a contour plot (BCEA). Neither provides a combination of both these plot elements.

We therefore developed a novel open source graphical presentation of PSA results, incorporating relative density and probability contours, overcoming both overdrawing and outlier overestimation. The method is independent from modelling software and relies only on an import of PSA results in.csv format. We call this new PSA output presentation the Relative Density plot (PSA-ReD).

The aim of this paper is to illustrate the concept and functionalities of the PSA-ReD plot based on its application to two real-world case studies. We also provide the R-code designed for direct application to any user’s own research outputs together with a user manual on GitHub (details provided after blinded review, reference 14).

## Methodology

### Relation to traditional cost-effectiveness plane

**Fig. 1:**
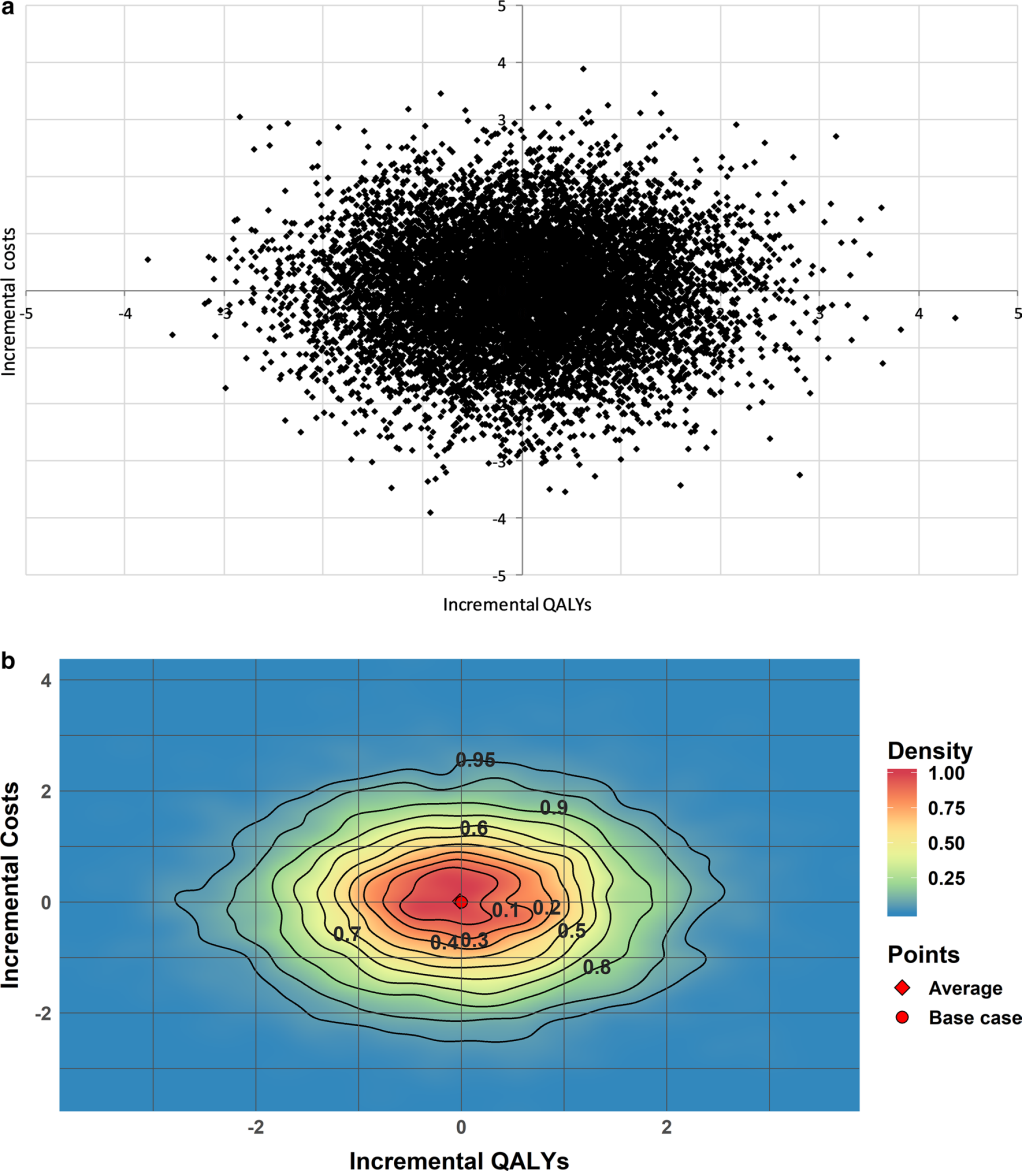
**a** Traditional scatterplot displaying PSA results. **b** New graphical presentation of PSA using the Relative Density plot (PSA-ReD) using a bivariate normal distribution with mean = 0.0, sd = 1.0, 10,000 iterations and 1000 bins. *PSA* Probabilistic Sensitivity Analysis

A traditional PSA output is a two-dimensional black and white scatter plot presented on a CE-plane (Fig. 1a). The PSA-ReD plot (Fig. 1b) combines a multi-colored density plot (Additional file 1: Fig. S1a) and a contour plot (Additional file 1: Fig. S1b). The combination of these two plots allows the reader to identify and distinguish high density areas using a color scale, as well as a quantification of the point estimate density within the CE-plane, thus visualizing the information that remains hidden in the traditional scatter plot. This increases the information that can be understood from the scatter plot and improves understanding of the parameter uncertainty which a PSA is aimed to address.

To explain how the PSA-ReD plot works, we provide a simulated example in Fig. 1b. It assumes a model with only two standard normally distributed parameters (mean = 0 and standard deviation = 1) that define the incremental costs and incremental QALYs.

In Fig. 1a, the base case would be at zero incremental costs and zero incremental QALYs. Though we can see that the borders of the area are less densely populated, it is unclear how the density of iterations is spread over the populated area. If instead we look at the PSA-ReD plot in Fig. 1b, it becomes clear that the density is evenly spread around the base case, as would be expected for this normally distributed data. Additionally, the contours give insight into the spread of the iterations. In this case, the area containing 95% of the iterations will approximate that of a 95% confidence interval because we used normal distributions. A bivariate normal distribution is distributed according to the χ^2^-distribution with two degrees of freedom [14]. Taking the square root of the critical value for the 95% confidence interval (5.99), results in the area borders of the confidence interval (2.45). This is clearly shown by the contours in the PSA-ReD plot. In general, the probability that the values for two variables within a joint distribution together fall in any area of their two dimensions is given by the volume (or cumulative probability) under the density function above that area. This is exactly what the PSA-ReD method calculates, as is explained in the next section.

### The density plot

The density plot (Additional file 1: Fig. S1a) could be interpreted as a two-dimensional histogram. Like a traditional one-dimensional histogram, the two axes are divided in sections. These sections on both axes divide the two-dimensional space in distinct rectangular regions. Then, as in a one-dimensional histogram, the number of data points per region is counted and transformed to present the relative frequency using a color scale. Low density is presented by a green to blue scale and high density is presented by a yellow to red scale.

When using a histogram, the choice of the anchor point of the plot area (i.e., the range and starting points of the axes) has influence on the graphical outcome [15]. This effect would be most pronounced in a one-dimensional histogram with relatively few datapoints: Shifting the x-axis would change the histogram as, by chance, the number of datapoints falling within each bin would differ with each x-axis shift. Crucially, the underlying data remains the same and this bias would also be present in two-dimensional histograms [15]. To overcome this, bivariate kernel density estimation (kde) is used as it provides a more accurate representation of the probability density [15]. Instead of counting the number of data points per rectangular section, each data point is surrounded by a kernel which are summed to yield the kernel density estimate. Each data point is thereby smoothed over a small surrounding area (data kernel) instead of being a single data point [15]. The size of this area is determined by the data as explained in the ‘technical aspects’ paragraph. Consequently, the way the resulting plot looks does not depend anymore on the size of any bins over the x-axis or y-axis.

### The contour plot

The PSA-ReD plot combines the density plot with a contour plot (Additional file 1: Fig. S1b). The contours indicate the boundaries of regions with a specific cumulative probability. This cumulative probability is calculated by summing the area density estimates (retrieved from the kernel estimation method). For this summing of density estimates to cumulative probabilty, densities are sorted from high density to low density with the summing starting from the highest density. These cumulative probabilities are then mapped to a range of 0 to 1. In this way, the total density in the plot area sums to one, reflecting the cumulative probability. A contour line is then drawn joining areas with specific pre-specified values of cumulative probability. For each individual plot area in the PSA-ReD, two values are now available: the density per area and the cumulative probability that is reached per area. A contour line is then drawn joining areas with specific pre-specified values of cumulative probability. These values can be chosen by the user (e.g. 0.1, 0.5, 0.95).

When there are multiple, disjointed high (or low) density-areas, it is possible that separate contours with equal cumulative probability values are drawn over these separate areas. For example, when there are separate high-density areas that together amount to 50% of the data points, seperate contour-lines with the value of 0.5 would be drawn around both high density values.

### Hardware and software

The script for the PSA-ReD plot was developed and tested using R version 3.5.1 and Rstudio version 1.1.453 (R Core team [11, 16]). For our analyses, we used a standard consumer grade personal computer (Dell OptiPlex 9020). In the technical appendix (Additional file 2), we provide detailed information on the hardware and software used.

The R script that we used is available in a GitHub repository [17]. We adhered to Google’s R Style guide and provide step-by-step guidance using comments embedded in the script [18]. The R script is licensed under the GNU General Public License v3.0 [19]. In short, this means that users are free to run, study, share and modify the software. The license dictates, among other things, that the software (or derivative work) must be open source and that derivative work must be published using the same license [19]. This guarantees that our project can be used and optimized by anyone whilst ensuring that it remains open to all.

### Technical aspects of plot generation in R

In R, we use the kde2d function from the Modern Applied Statistics with S (MASS) package to perform the aforementioned kernel density estimation [20]. In essence, the outcome of kde is a density value per area of a prespecified size, comparable to the number of data points within each bin in histograms. Detailed information is provided in the work by Silverman and in the documentation of the MASS package [20, 21]. As these density values are very small and hard to interpret, we normalize these values by taking the reciprocal of the maximum density value to yield values ranging from 0.0 to 1.0. With these, we generate an easy to interpret plot with a scale from 1.0 (highest density) to 0.0 (lowest density).

The kde2d function has, besides the x and y values, two arguments that influence the kde. These are n (the number of bins in each dimension) and h (the bandwidth that determines the level of smoothing). The number of bins defines the number of sections on each axis. The total number of areas within the resulting plots is therefore horizontal bins * vertical bins (e.g. 100*100 = 10,000). An easy analogy of these areas would be to regard them as pixels, the bins then determine the resolution in both directions. This pixel-analogy only reflects to the number of underlying bins. As we outline in the Additional file 2 (Technical Appendix) regarding the saving of plots, the actual resolution of the figures can be specified and is irrespective of the number of bins used. As the number of bins can be interpreted as the resolution of the figure, a larger number of bins produces a more precise figure. However, increasing the number of bins also increases computation time which means a balance must be struck.

In Additional file 1: Fig. S2, we present the influence of different bin sizes. Using 50-500 bins (Additional file 1: Fig. S2a, b), yields a density gradient that is not smooth and may appear like the image is pixelated. With 1000 bins (Additional file 1: Fig. S2c), the image is smooth, no pixilation can be identified and all the computation is performed within 1 minute on the aforementioned consumer grade computer. With more bins (2000, Additional file 1: Fig. S2d), the image does not get smoother but it does lead to increased RAM usage and computation time. Another consequence of a lower number of bins (as in Additional file 1: Fig. S2a) is that due to the lack of smoothing, the contours are placed different from when more bins are used. Essentially, with fewer bins, the contours are very rough. We therefore recommend using 1000 bins and have used this number of bins in all figures throughout the manuscript, unless otherwise stated.

The h argument of the kde2d function determines the bandwidth of the kernel areas. It can be interpreted as the size of the kernels that is applied when converting each data point to a data kernel. We have chosen to leave this at the default setting where the bandwidth is automatically selected based on the data by the well-established MASS package (specifically, the bandwidth.nrd function) [20]. This guarantees generalizability of results.

### Number of PSA iterations

As in any PSA, it is preferred to run as many iterations as necessary to reach model convergence [6]. We explored the influence of the number of iterations used by varying this between 1000 and 100,000 iterations, as presented in Additional file 1: Fig. S3. As RAM usage and computation time increases when more iterations are used, we recommend using a maximum of 10,000 iterations. Running the script with 10,000 iterations takes a maximum of 1 min. In all figures throughout this manuscript, we have used 10,000 iterations unless otherwise stated.

### User modifications

Other parameters that can be altered by the user are contour levels, axis-, legend- and plot titles, font sizes and font types. In the supplied script, it is explained how and where this can be done. Apart from these cosmetic changes, we provide means to zoom on a particular area of the plot and generate a new plot from that specific area. Additional file 1: Fig. S4 displays this zooming capability. We also provide a feature that allows users to plot willingness-to-pay (WTP) thresholds in the PSA-ReD plot, as well as plotting the average incremental costs and incremental effects for the PSA and the results of the deterministic base case scenario. The technical appendix (Additional file 2) provides in-depth explanations on the use of the various features described above.

### Case study demonstration

To demonstrate the novel graphical presentation, the concept was applied to two exemplary case studies. These real-world case studies were selected as a convenience sample as we needed access to the raw PSA results. To increase transparency, we opted for published case studies. The two selected cases each show a different pattern within the PSA results. Both patterns are commonly seen in economic evaluations. The first real-world case study assesses the influence of three characteristics (cost, specificity and sensitivity) on cost-effectiveness of a hypothetical pharmacogenomic test for prevention of angiotensin-converting enzyme inhibitor induced angioedema (denoted as ‘eHTA study’) [22]. The second real-world case study used a three-state partitioned survival model to investigate cost-effectiveness of periodic therapeutic drug monitoring of endoxifen levels in breast cancer patients (denoted as ‘TDM study’) [23].

## Results

### eHTA case study

**Fig. 2:**
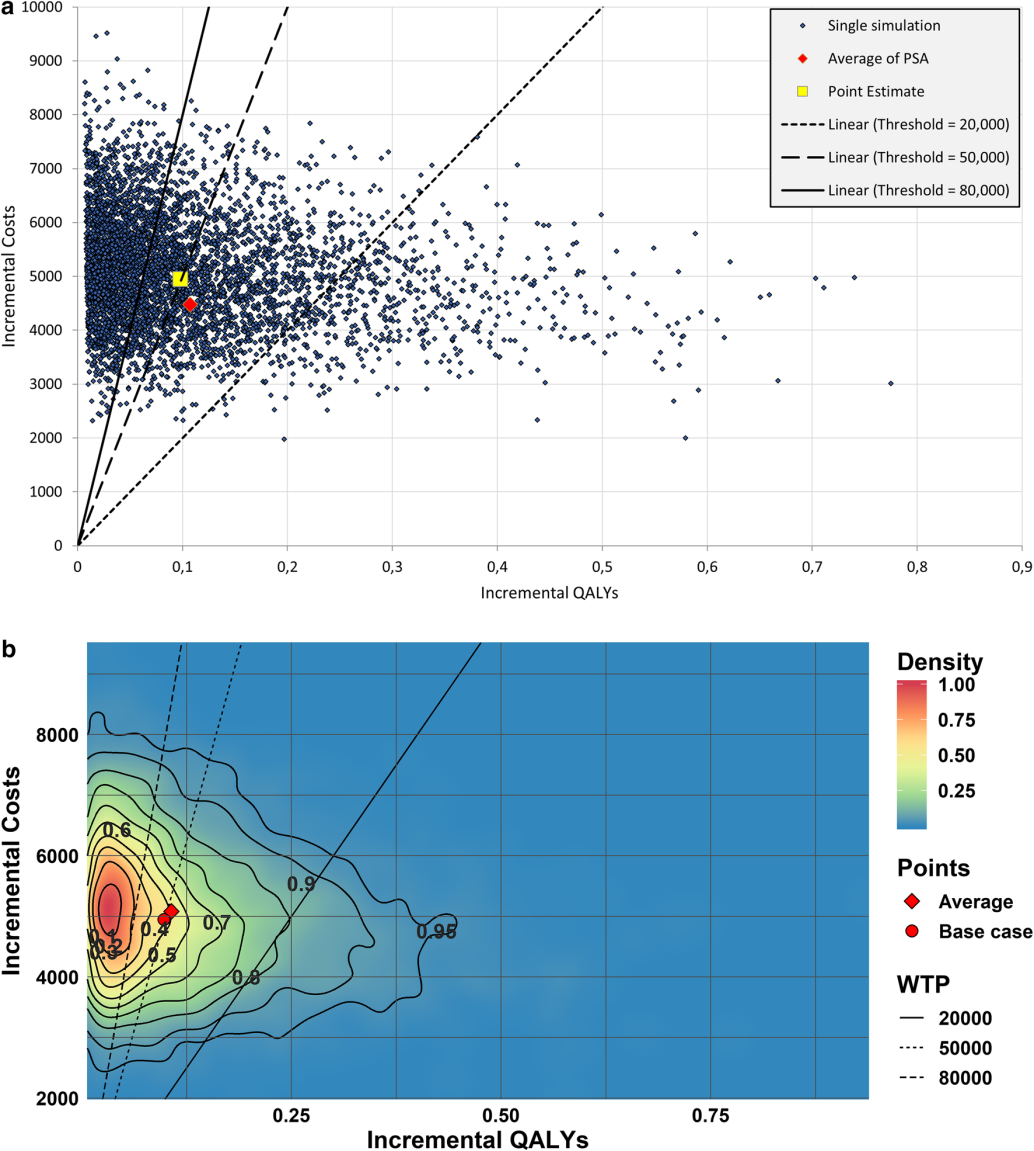
Probabilistic sensitivity analysis (PSA) output of eHTA-study. **a** Traditional presentation. **b** New graphical presentation of PSA using the Relative Density plot (PSA-ReD)

The results of the PSA of the eHTA study are presented in Fig. 2. This figure shows the PSA results both in traditional presentation (2A) as well as via the PSA-ReD plot (2B). The figures are both based on 5000 PSA iterations, as this reflects the number of iterations in the published paper [22]. The classic CE-plane implies more spread due to a small number of iterations that generate relatively high incremental QALYs. However, the PSA-ReD plot shows these are extremely infrequent and fall outside the contour area that includes 95% of the iterations. Additionally, 10% of all iterations appear within an area of approximately 0.05 incremental QALYs (0.0 –0.05) and 1000 incremental euros (4000–5000). Particularly interesting is that the base case falls well outside this most dense area. This is contrary to what would be expected in a PSA as generally, the most likely outcome for the incremental cost-effectiveness ratio (ICER) based on the individual distributions of parameters is close to the base case. Thus, one would expect the highest density area to be surrounding the base case.

However, when one of the model parameter distributions is skewed (i.e. a beta or gamma distribution), the resulting average of all PSA samples will, by definition, not lie on the point of highest density as the average will lean towards the tail of the specific distribution. In certain parameterizations of the beta and gamma distributions (e.g. when α < 1 and β > α), the base case value will not be the value with the highest probability density of that specific distribution. Instead, the value of 0 will have the highest probability density. In the eventual PSA-ReD plot, this effect attenuates the area of highest density away from the base case towards 0 as is especially apparent in this case study. This information cannot be interpreted from the traditional CE-plane. Therefore the PSA-ReD plot can provide modelers with information regarding model behavior.

A.csv datafile with the incremental QALYs (x-values) and incremental costs (y-values) of the eHTA PSA results is provided in the GitHub repository to allow the reader to recreate the PSA-ReD.

### TDM case study

**Fig. 3:**
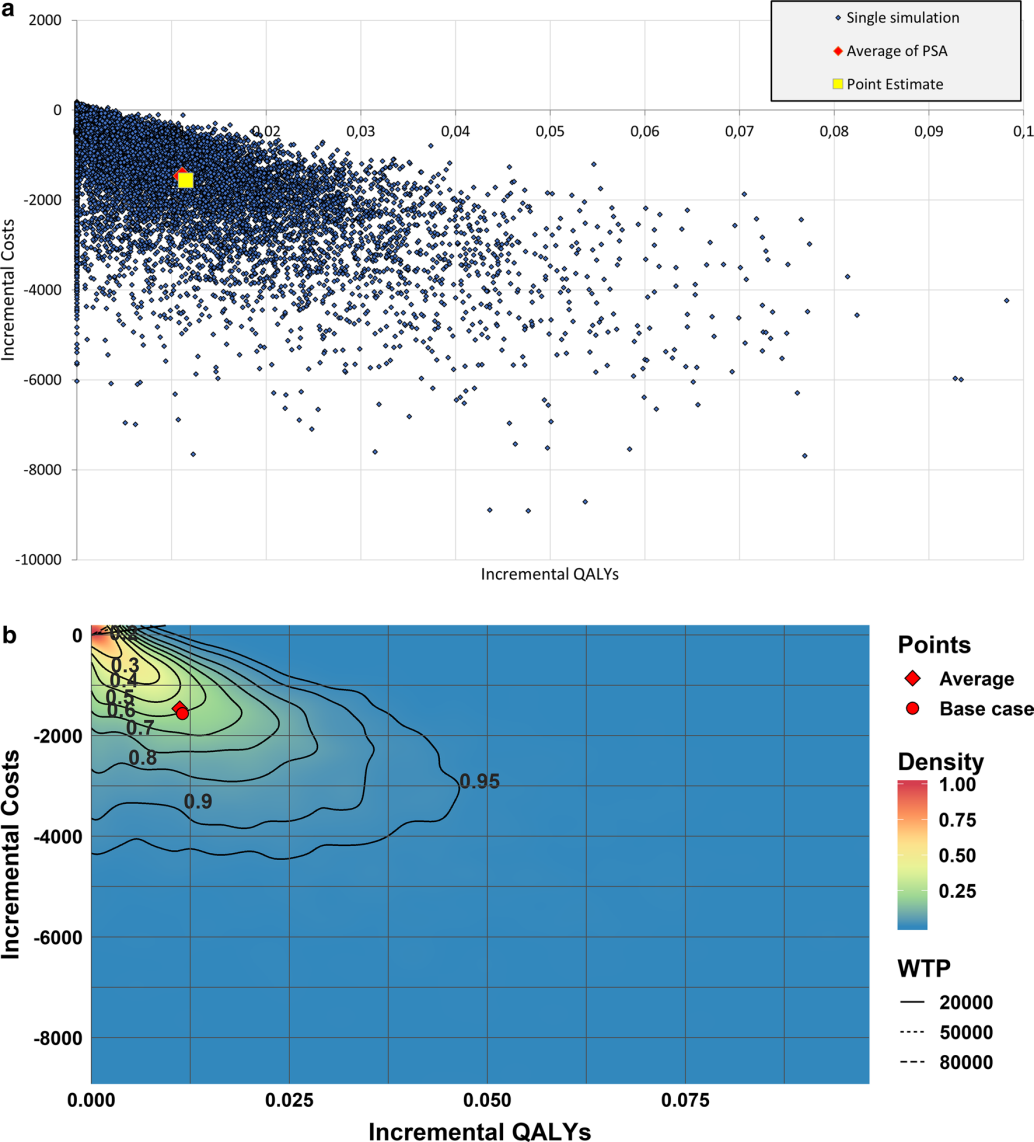
Probabilistic sensitivity analysis (PSA) output of TDM-study. **a** Traditional presentation. **b** New graphical presentation of PSA using the Relative Density plot (PSA-ReD)

Figure 3 shows the PSA results from the TDM case study both in traditional presentation (3A) as well as via the PSA-ReD plot (3B). Both figures are based on 10,000 PSA iterations. Density in the classic CE-plane is not interpretable but suggests a relatively high density around the base case and in the upper left corner of the plane. Additionally, there seems to be accumulation of iterations along the Y-axis. The PSA-ReD plot more precisely clarifies the high density that is found within the small area near the origin. Additionally, the relatively high density suggested by the CE-plane around the Y-axis is put into perspective by the PSA-ReD plot, clarifying that these scenarios are relatively infrequent. The PSA-ReD plot nevertheless provides the user with the information that some iterations accumulate around the x-axis through the contours that end up going into the y-axis rather than all curving toward the high highest density area.

## Discussion

Through the normal distribution example in the methods section, we demonstrated the application and interpretation of the relative density estimation function and the applied normalization of the values on which the PSA-ReD plot is based. In two real-world case studies, we demonstrated how the PSA-ReD plot provides more insight into the relative density and cumulative area probabilities. Thus, the PSA-ReD plot provides visual information that is not provided by the traditional PSA scatter plot within the CE-plane nor by solely a density plot or a contour plot. Another benefit of the PSA-ReD plot is that it provides very clear insight that uncertainty distributions may be non-linear. In both case studies, the highest density areas were not centered around the base case or the PSA average, in fact the base case and PSA average values lied outside the 0.4 cumulative probability contour around the most dense area for both case studies. This information is valuable to modellers as it provides them with insight about model functioning. If the extent of the resulting skew cannot be readily explained, possibly the model does not function as intended.

The traditional scatterplot is often accompanied by a cost-effectiveness acceptability curve and/or frontier. A cost-effectiveness acceptability curve will provide decision-makers with information regarding the likelihood of an intervention being cost-effective given certain WTP-thresholds. PSA-ReD, as traditional scatterplots, does not provide the decision-maker with this information, as the contours show cumulative probability around the base case rather than probability in relation to a threshold. PSA-ReD provides modellers with additional insight into the distribution of uncertainty in the PSA as opposed to a traditional scatterplot, but does not provide the information a CEAC provides. Therefore, PSA-ReD should be complemented with cost-effectiveness acceptability curves as would also be the case for the traditional scatterplot.

The benefits of the PSA-ReD plot over the traditional scatter plot are evident. The accumulation of PSA results within certain areas of the cost-effectiveness plane can often not be interpreted by the traditional scatter plot. The PSA-ReD plot not only clearly visualizes the location of these high-density areas, it also provides a quantification of the proportion of PSA iterations within these areas. Additionally, inappropriate significance could be attributed to relatively infrequent PSA iterations in the traditional scatter plot. The PSA-ReD plot diminishes this effect. Another benefit of the PSA-ReD plot is that it more appropriately reflects the distribution of uncertainty in the model as this distribution is in essence continuous. The PSA approximates this distribution by providing many iterations, but the PSA-ReD plot transforms these iterations into a continuous uncertainty distribution (through kernel density estimation), which is a more appropriate representation of model uncertainty.

Modellers should not be hampered by the current and clear graphical limitations of the scatterplot. The PSA-ReD plot provides modelers with increased insight into the relation between all input parameter distributions and the subsequent distribution of model outcomes. This can serve as an additional validation to confirm the model works as intended. Besides additional validation, PSA-ReD can help the modeler identify nuances that might be overlooked or hidden in the traditional PSA, such as the extremely high density area presented in the TDM case-study. Our two case studies furthermore demonstrated some benefits of PSA-ReD for models that have skewed PSA distributions, but these benefits would likely be greater for highly non-linear models as is for example often seen within infectious disease modelling. In general, it is becoming increasingly important to critically review the distributions of uncertainty within cost-effectiveness models due to its increased relevance in light of drug approval processes that inevitably come with increased uncertainty, such as conditional approval [24, 25]. We believe that maximum insight in the complex interplay between model structure, parameters and parameter distributions allows the modeler to make better decisions.

Currently, R packages exist that provide the option for plotting density figures. However, the corresponding documentation is typically hard to decipher and interpret for inexperienced users, the packages lack abilities for user adjustments and the packages typically require the user to perform all model syntax according to the construct of these packages. The heemod package for example, is a package specifically designed for cost-effectiveness analysis [12]. Though it does provide the option of generating a density plot, this does not generate contours nor does it provide user options such as the plotting of WTP-thresholds. To display results in a density plot using the heemod package, users need to understand and use the heemod package syntax. Another example is the BCEA package which has a variety of graphical capabilities but also requires users to use the specific syntax [13]. An alternative previously described approach to illustrate areas with a specific cumulative probability is the ellipse, for example implemented as a 95% confidence ellipse by Pradelli et al. and as an option in the software suite TreeAge [26, 27]. This approach has several weaknesses. First, it assumes the underlying distribution is elliptical. This would be correct for our normally distributed example but is clearly not suitable for the two real-world case studies. Our non-parametric density estimation does not rely on this assumption. Second, there is no single or clear method on how to generate the ellipse which potentially limits generalizability. Indeed, the example of Pradelli et al. does not describe the methodology used to generate their ellipse. Third, there is no readily available and generic implementation of the ellipse methodology in for example Excel or R, so this functionality could only be available if the health-economic model is built within a specific proprietary software package. Our approach to the PSA-ReD plot is specifically designed to combine a density plot with a contour plot in one figure and to be used with any model and any software, as long as the user is able to extract the PSA x- and y-values and save them as an.RData, Microsoft Excel or.csv file which thereafter can be imported into R using our script. This facilitates maximum applicability of the PSA-ReD plot code. It does not matter in which software program the model has been constructed or even what type of probabilistic analysis has been performed.

To facilitate the use of our method we provided a step-by-step tutorial on GitHub to generate the PSA-ReD plot based on PSA results from any user’s own research (J. W. Geenen, 2018/2019). This tutorial is designed to also accommodate users with very basic R knowledge. Additionally, generating a variety of PSA-ReD plots is easier and quicker than generating multiple attractive plots in Excel.

For modelers who do not wish to use or explore R, it is possible to generate a 2D histogram with colors within Excel. This approximates the density part of the PSA-ReD plot but lacks the kernel density estimation and contours. It also does not provide the option for adding WTP thresholds nor any of the user options to adjust the figure to make it more visually attractive. An Excel file including the Visual Basic Application syntax can be requested from the authors. However, we highly recommend using R for PSA-ReD generation. The R script provides the option to construct only a contour or only a density plot.

The PSA-ReD script bases the size of the plot exactly on the minimum and maximum values of the PSA iterations in the dataset. This means that four PSA points (or less if they define a corner) lie exactly on the borders of the PSA-ReD figure. As plots usually have some space around the minimum and maximum values, this may make the initial interpretation of the total range slightly harder, but we believe that this yields the best insight into the (distribution of) high density areas as the plot size is kept as small as possible.

The provided R script provides a selection of user options to modify the generated plot. These options and settings are aimed at providing all the functionalities that users of the current scatter plots require. Though experienced R users may be able to further customize the script, novel users are encouraged to apply the options provided in this paper to ensure generalizability of the PSA-ReD plot generated by different users.

## Conclusion

The proposed PSA-ReD plot facilitates intuitive visual interpretation of information included in PSA results that cannot be derived from the traditional scatter plot. Specifically, the PSA-ReD plot provides truly additional quantitative information on the relative density of PSA outcomes. Furthemore, it gives insight in the cumulative probability of PSA iterations within predefined areas of the cost-effectiveness plane. The PSA-ReD plot can thus, compared to the traditional scatter plot, provide a more detailed presentation of the highest-density areas, quantify their cumulative probability, and is not prone to over-emphasis of infrequent PSA iterations.

These improvements allow the modeler to gain a better and an unbiased insight in the underlying dynamics of model structure, parameters and parameter distributions reflected in the uncertainty around the point estimate for the cost-effectiveness in a PSA-ReD plot. These insights can be leveraged for better model validation and development. PSA-ReD should not be seen as a completely new technology but instead as the the next iteration in the development of PSA as a means to gain insights in model functionality and outcome uncertainty.

## Supplementary information

**Additional file 1:** Additional figures.

**Additional file 2:** Technical appendix.

## Data Availability

All data, scripts and methods used are openly accessible on a GitHub repository (https://github.com/joostgeenen/PSA-ReD). The script used to generate the PSA-ReD plots is licenced under GNU General Public License, allowing the use and adaptation of the script, as long as these derivative materials remain open-source and publicly available.

## References

[1] Adalsteinsson E, Toumi M (2013). Benefits of probabilistic sensitivity analysis – a review of NICE decisions. Journal of Market Access & Health Policy.

[2] Briggs AH, Weinstein MC, Fenwick EAL, Karnon J, Sculpher MJ, Paltiel AD (2012). Model parameter estimation and uncertainty analysis: a report of the ISPOR-SMDM modeling good research practices task force working group-6. Med Decis Making.

[3] NICE. (n.d.). Guide to the processes of technology appraisal Process and methods [PMG19]. 27905710

[4] Zorginstituut Nederland (ZIN), Staal, P., Heymans, J., Ligtenberg, G., Derksen, J., & Couwenbergh, B. (2014). *Beoordeling stand van de wetenschap en praktijk*. 90.

[5] Briggs A, Sculpher M, Buxton M (1994). Uncertainty in the economic evaluation of health care technologies: the role of sensitivity analysis. Health Econ.

[6] Hatswell AJ, Bullement A, Briggs A, Paulden M, Stevenson MD (2018). Probabilistic sensitivity analysis in cost-effectiveness models: determining model convergence in cohort models. PharmacoEconomics.

[7] Baio G, Dawid AP (2015). Probabilistic sensitivity analysis in health economics. Stat Methods Med Res.

[8] Drummond, M. F., Sculpher, M. J., Torrance, G. W., O’Brien, & Stoddart, B. J. and G. L. (2005). Methods for the economic evaluation of health care programmes. In *Oxford: Oxford University Press* (3rd ed., Vol. 3). Oxford University Press.

[9] Cleveland William S (2012). The Many Faces of a Scatterplot. Theory and Method.

[10] Gleicher AM (2013). Splatterplots: overcoming Overdraw in Scatter Plots. NIH Public Access.

[11] R Core team. (2015). R: A language and environment for statistical computing. R Foundation for Statistical Computing.

[12] Filipovic-Pierucci, A., Zarca, K., & Durand-Zaleski, I. (2016). Markov Models For Health Economic Evaluation Modelling In R With The Heemod Package. In *Value in Health* (Vol. 19, Issue 7, p. A369). 10.1016/j.jval.2016.09.133.

[13] Baio, G., Heath, A., & Berardi, A. (2017). *Bayesian Cost*-*Effectiveness Analysis with the R package BCEA*. Springer Nature.

[14] Davies, H. M., & Hope, K. (2007). Methods of Multivariate Analysis. In *The Mathematical Gazette* (3rd ed., Vol. 56, Issue 395). Wiley. 10.2307/3613737.

[15] April, S. (2003). Density Estimation for Statistics and Data Analysis Chapter 1 and 2. In *Most* (p. 8).

[16] RStudio. (2011). RStudio: Integrated development environment for R (Version 0.97.311). In J. Wildl. Manage. (Vol. 75, Issue 8, pp. 1753–1766). RStudio, Inc. 10.1002/jwmg.232.

[17] Geenen, J. W. (2019). GitHub repository for the PSA-ReD scripts and manuals. [R]. https://github.com/joostgeenen/PSA-ReD (Original work published 2018).

[18] Google. (2013). Googles R Style Guide (pp. 1–6).

[19] Free Software Foundation Inc. (2007). *GNU General Public License v3.0*.

[20] Venables WN, Ripley BD (2002). Modern Applied Statistics with S. Springer.

[21] Silverman, B. W. (1986). Density Estimation for Statistics and Data Analysis. Chapman and Hall/CRC.

[22] Geenen J, Baranova E, Asselbergs F, de Boer A, Maitland-van der Zee A, Hovels A (2016). Early HTA in Pharmacogenomics: a Case Example in Cardiovascular Drugs. Value in Health.

[23] van Nuland M, Vreman RA, Ten Ham RMT, de Vries Schultink AHM, Rosing H, Schellens JHM, Beijnen JH, Hövels AM (2018). Cost-effectiveness of monitoring endoxifen levels in breast cancer patients adjuvantly treated with tamoxifen. Breast Cancer Res Treat.

[24] Vreman RA, Geenen JW, Hövels AM, Goettsch WG, Leufkens HGM, Al MJ (2019). Phase I/II Clinical Trial-Based Early Economic Evaluation of Acalabrutinib for Relapsed Chronic Lymphocytic Leukaemia. Applied Health Economics and Health Policy.

[25] Vreman RA, Naci H, Goettsch WG, Mantel-Teeuwisse AK, Schneeweiss SG, Leufkens HGM, Kesselheim AS (2020). Decision Making Under Uncertainty: comparing Regulatory and Health Technology Assessment Reviews of Medicines in the United States and Europe. Clin Pharmacol Ther.

[26] Pradelli L, Povero M, Muscaritoli M, Eandi M (2015). Updated cost-effectiveness analysis of supplemental glutamine for parenteral nutrition of intensive-care patients. Eur J Clin Nutr.

[27] TreeAge Software, Inc. (2017). TreeAge Pro 2017 R2 User’s Manual. 795.

