# Supplementary material for: Increasing the information provided by probabilistic sensitivity analysis: The relative density plot
Source: Cost Eff Resour Alloc. 2020 Nov 30;18:54. doi: 10.1186/s12962-020-00251-7 (PMC7706250; doi:10.1186/s12962-020-00251-7)
Supplement: Supplementary file 1 — Additional file 1: Additional figures. [file 12962_2020_251_MOESM1_ESM.docx]

**Additional file**

**Additional file: Figure S1: Figures of Components of PSA-ReD**

**
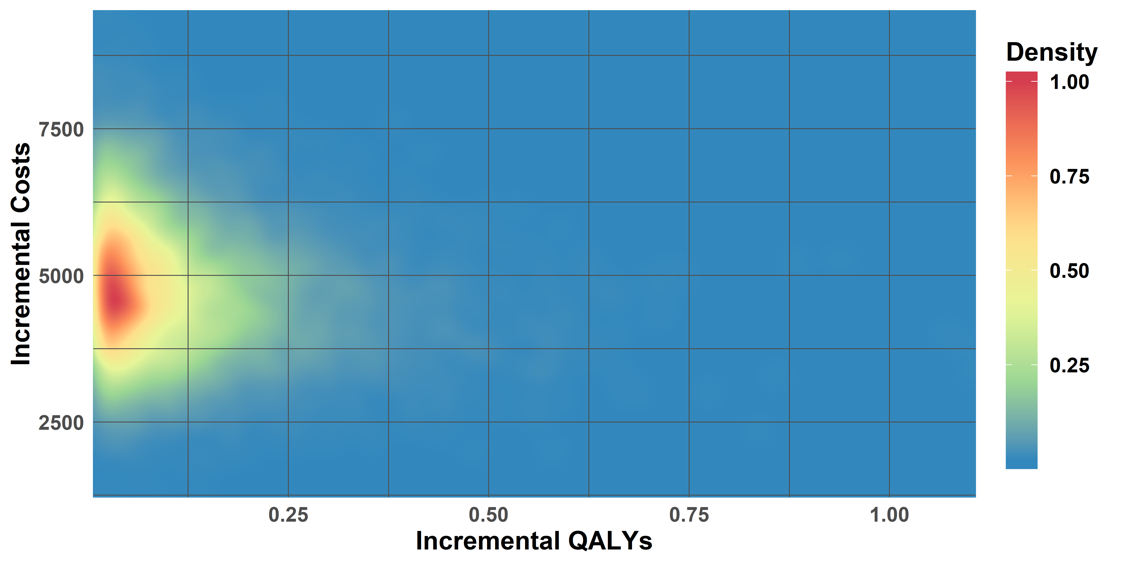
**

**Fig. S1a** Figure with only the density rendered, generated using 1000 bins and 10,000 PSA iterations

**
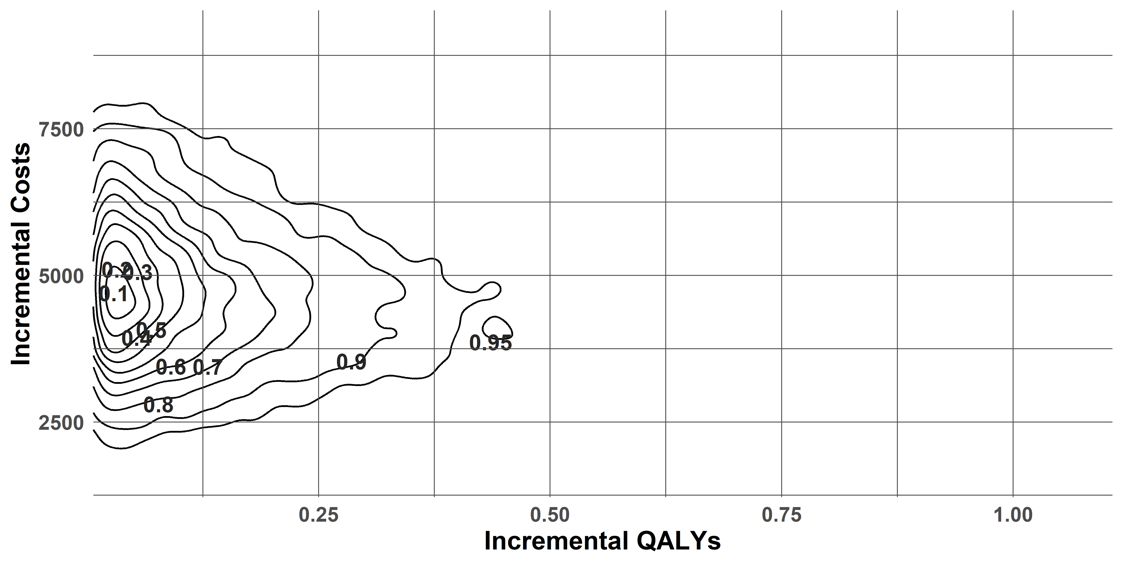
Fig.S1b** Figure with only the contours rendered, generated using 1000 bins and 10,000 PSA iterations

**Additional file: Figure S2: Influence of bin size on PSA-ReD plots**


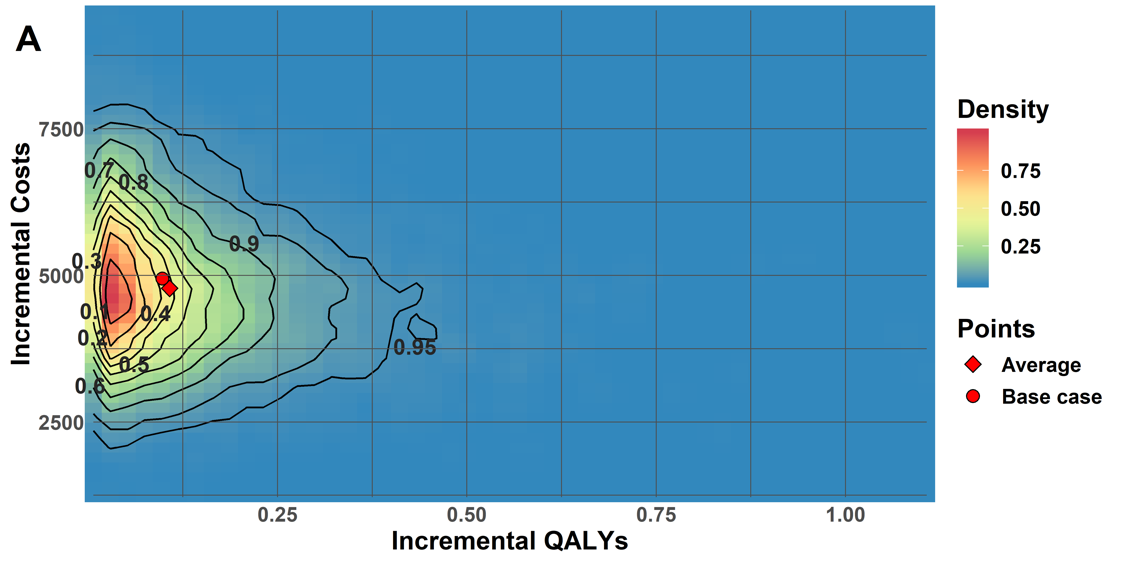


**Fig.S2a** Bin size = 50, 10,000 PSA iterations


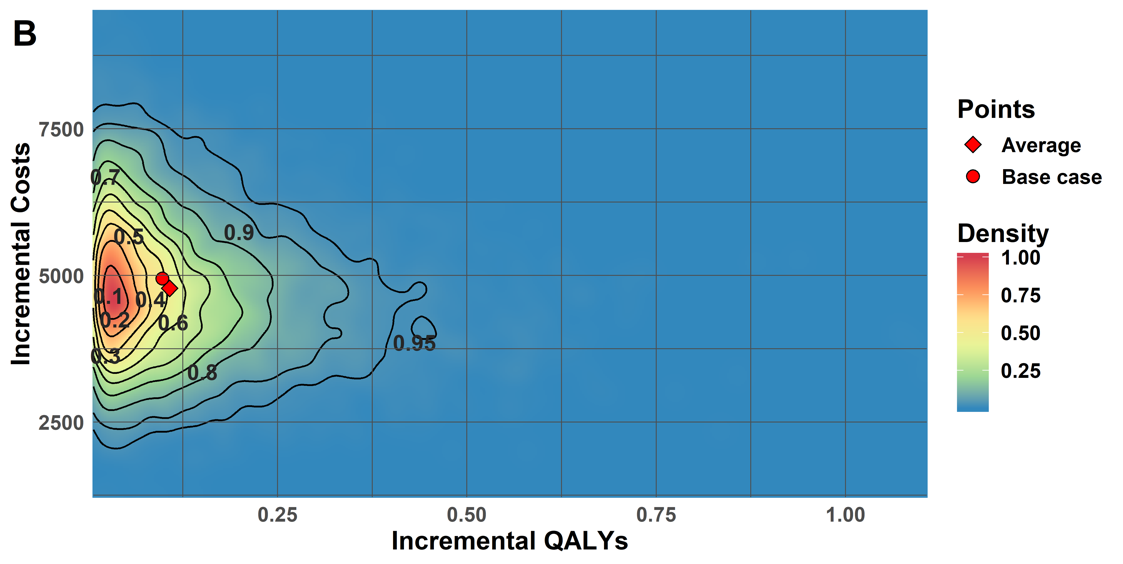

**Fig.S2b** Bin size = 500, 10,000 PSA iterations


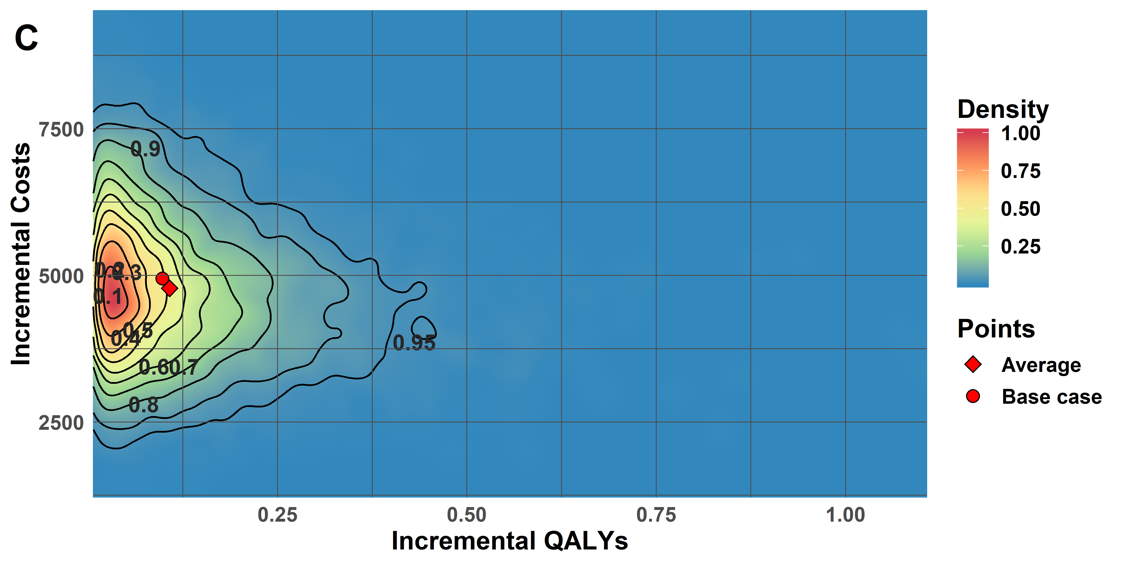


**Fig.S2c** Bin size = 1000, 10,000 PSA iterations


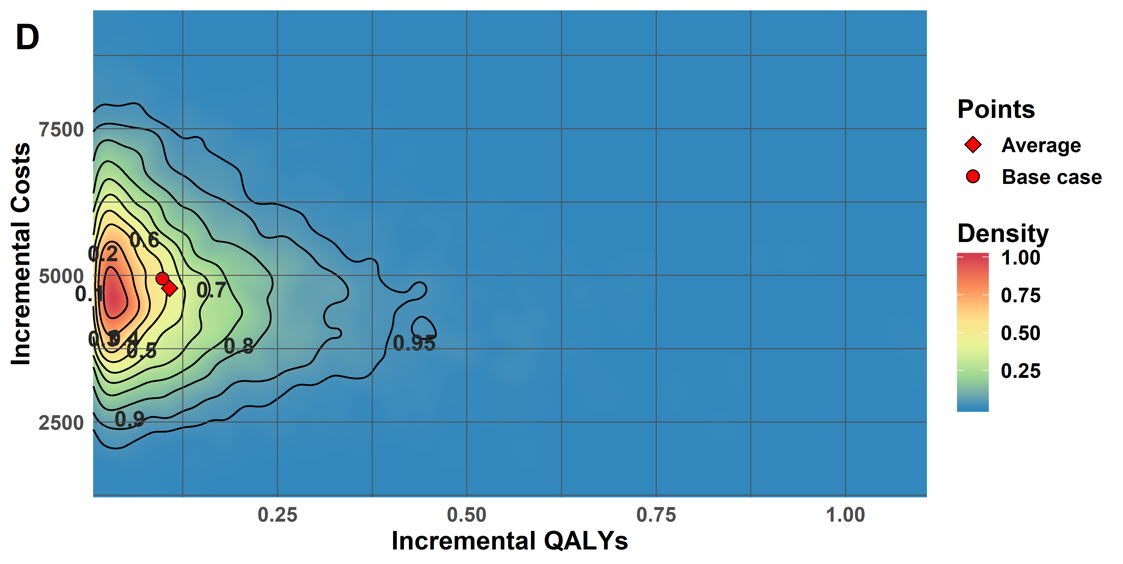


**Fig.S2d** Bin size = 2000, 10,000 PSA iterations

**Additional file Figure S3: Influence of number of PSA iterations on PSA-ReD plots**

**
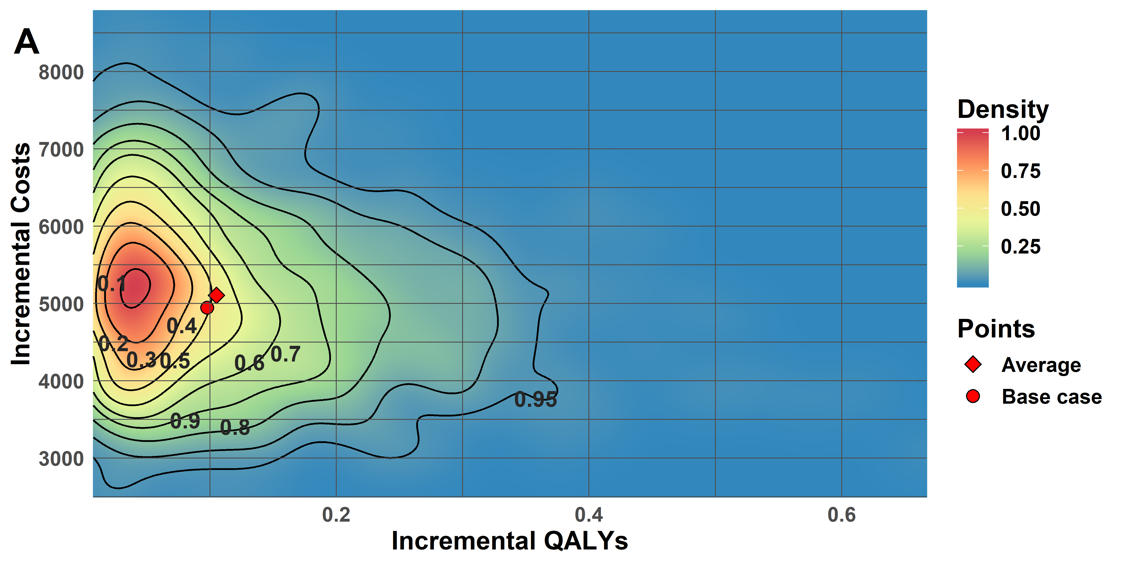
**

**Fig.S3a** Bin size = 1000, 1000 PSA iterations

**
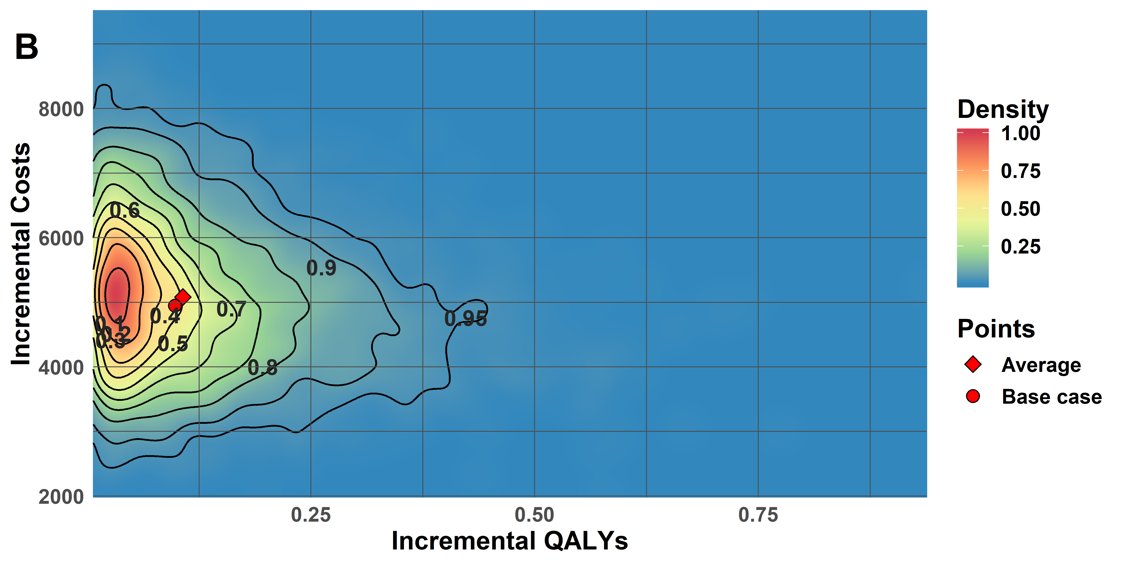
**

**Fig.S3b** Bin size = 1000, 5000 PSA iterations

**
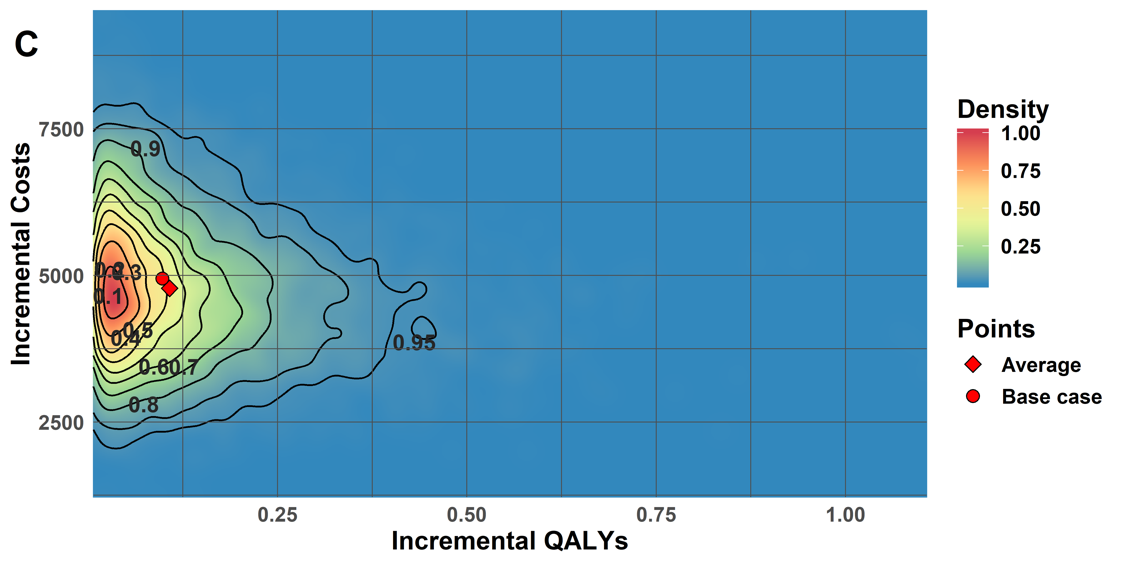
**

**Fig.S3c** Bin size = 1000, 10,000 PSA iterations

**
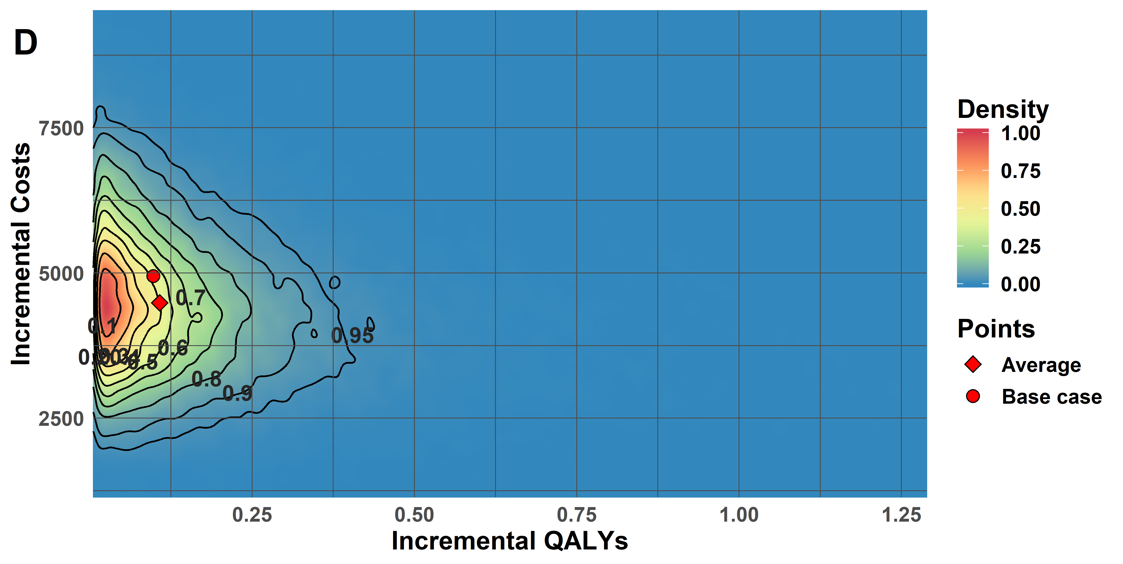
**

**Fig.S3d** Bin size = 1000, 100,000 PSA iterations

**Additional file Figure S4: Zoom functionality**

**
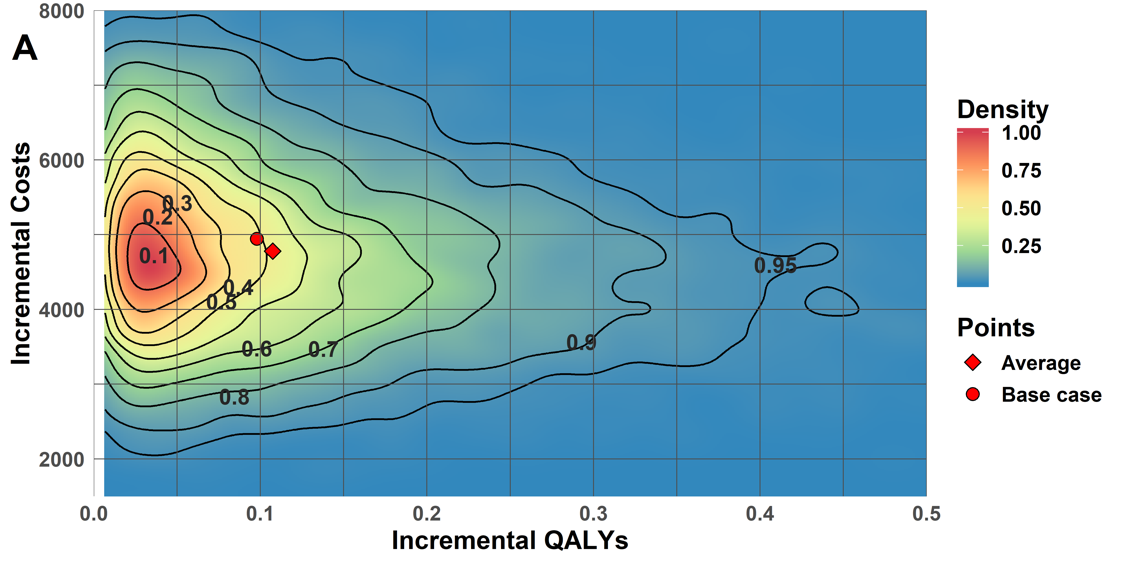
**

**Fig.S4** Zoomed PSA-ReD plot, clip = FALSE. Bin size = 1000, 10,000 PSA iterations. Compare this Figure to Supplemental Figure 2c or 3c and review the effect of the zoom functionality.

**Additional file: Figure S5: Influence of clip argument**

**
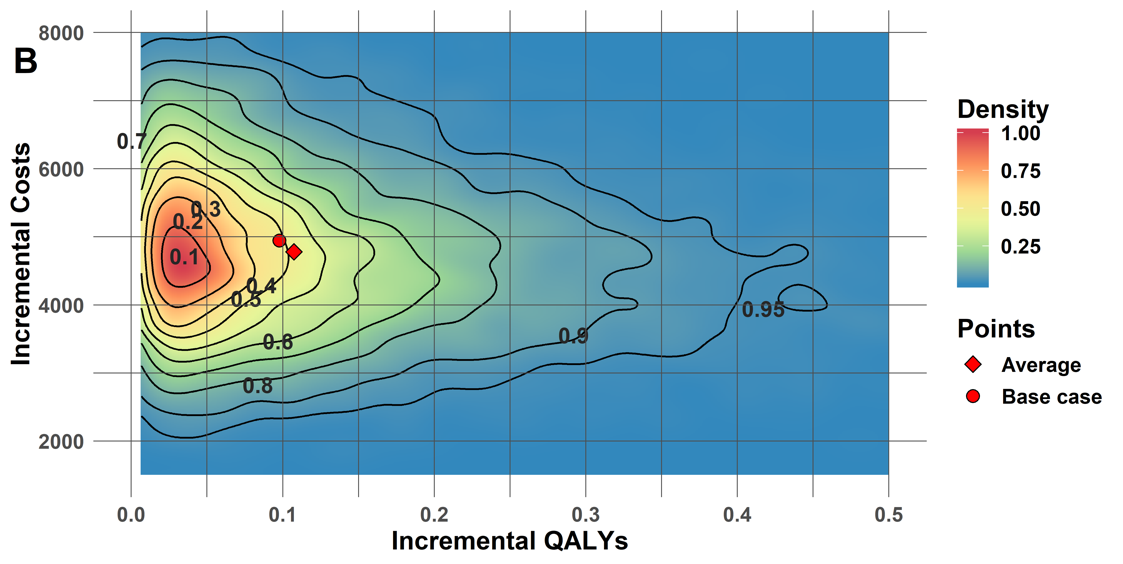
**

**Fig.S5** Zoomed PSA-ReD plot, clip = TRUE. Bin size = 1000, 10,000 PSA iterations. Compare this Figure to Supplemental Figure 4 and review the contour line labelling.
