# Supplementary material for: Increasing the information provided by probabilistic sensitivity analysis: The relative density plot
Source: Cost Eff Resour Alloc. 2020 Nov 30;18:54. doi: 10.1186/s12962-020-00251-7 (PMC7706250; doi:10.1186/s12962-020-00251-7)
Supplement: Supplementary file 2 — Additional file 2: Technical appendix. [file 12962_2020_251_MOESM2_ESM.docx]

**Technical Appendix**

Hardware and software used

Various packages exist for rendering plots and figures. We have selected ggplot2 as renderer as it is free, open source, very versatile, easy to interpret and very well supported [1]. Besides ggplot2 version 3.0.0, the following R packages were used: MASS version 7.3-50 to perform kde, directlabels version 2018.05.22 to add contour labels, grid version 3.5.1 and gridExtra version 2.3 for conveniently displaying and zooming the figures and reshape2 version 1.4.3 for data preparation [1-5]. The supplied script handles installing and/or loading of these packages. For our analyses, we used a standard consumer grade personal computer (Dell Optiplex 9020, Intel ® Core ™ i5-4590 CPU @ 3.30 GHz, 8.00 GB Random Access Memory (RAM), 500GB 7200RPM Hard Disk Drive, Windows 10 Enterprise © 2017 Microsoft Corporation).

Zooming

As the initial plot range is chosen on the maximum and minimum x and y values, this implies that some data points are removed when zooming in. We do however think that adding this option is justified as some PSA samples result in extreme x and y values that are of little relevance to the interpretation of the PSA as a whole. The likelihood of such an event is bigger when using a very large PSA sample (i.e. 100.000). A few extreme values will increase the plot size and may limit the interpretability of the area of interest. The implementation of zooming is chosen so that the plot itself is trimmed instead of trimming the underlying data. This conserves the underlying data structure and is analogous to simply selecting a small part of an existing larger figure. Users can use the zoom functionality by specifying a specific x and y range. Supplemental Figure 4 provides an example of the zoom functionality.

When zooming, users have to pay attention to whether the contour labels are clipped from the plot area. We have supplied a setting that can deal with this issue but it does require the user to evaluate their zoomed figure and choose the most appropriate figure. Supplemental Figure 5 provides an example of how clipping can affect the contour labels.

Saving

Figures can be saved using functions that are supplied in the script. The path and filename can be set by the user. Besides these settings, one can choose the resolution (as dpi) with which the figure is saved. We specifically chose the dpi as setting to alter the eventual figure size as it does not influence the relative sizes of the various figure elements.

When saving the figure, one can choose the figure to be saved exactly like it appears in the Rstudio “Plots” panel. As different users and computers could have differently sized “Plots” panels, we supply a means to specify the required width and height of the figures. This ensures that different users can easily generate the same PDP when using the same data and settings.

Plotting Willingness to Pay threshold

Willingness to pay (WTP) thresholds are regularly plotted in the traditional PSA scatter plot. We have built a feature that allows users to specify one or more WTP thresholds. If one wishes to plot these thresholds, the WTP thresholds should be stored in the vector “WTP.thresholds” which is present in the script.

**References**

1. Wickham H. ggplot2: Elegant Graphics for Data Analysis [Internet]. Springer-Verlag New York; 2016.

2. Venables W, Ripley B. Modern Applied Statistics with S [Internet]. New York: Springer; 2002.

3. Hocking T. directlabels: Direct Labels for Multicolor Plots [Internet]. 2018.

4. Auguie B. gridExtra: Miscellaneous Functions for “Grid” Graphics [Internet]. 2017.

5. Wickham H. Reshaping Data with the reshape Package. Journal of Statistical Software. 2007;21:1–20.
